# NIGERIA'S NATIONAL SECURITY STRATEGY AND THE ROLE OF SECURITY AGENCIES IN COMBATTING KIDNAPPING IN THE FEDERAL CAPITAL TERRITIORY ABUJA FROM 2021-2025

**DOI:** 10.12688/openresafrica.16586.2

**Published:** 2026-09-03

**Authors:** Aisha Annie Yahaya, Dimas Garba

**Affiliations:** 1 Political Science and International Relations, Nile University of Nigeria, Abuja, Federal Capital Territory, Nigeria

**Keywords:** Federal Capital Territory, Kidnapping, National Security Strategy, Security Agencies, Nigeria.

## Abstract

This article examined the escalating threat of kidnapping within Nigeria’s Federal Capital Territory (FCT) from 2021 to 2025. The objective is to critically evaluate the extent to which the 2019 National Security Strategy (NSS) has been effectively operationalized by relevant security agencies in addressing kidnapping. Mixed research methods, and the Routine Activity Theory and Institutional Capacity Theory were applied. Both theories illuminate how the convergence of motivated offenders, unsuitable guardianship, and institutional weaknesses sustains the kidnapping economy in Nigeria’s capital. Drawing on an original incident-level dataset of 167 documented abduction events in the FCT spanning 2021 to 2025, qualitative expert testimony from a senior security intelligence practitioner, and a comprehensive review of the theoretical and empirical literature, the study found that kidnapping incidents increased dramatically over this period from 21 incidents and 62 victims in 2021 to a peak of 50 incidents and 247 victims in 2023 before a partial numerical decline in 2024 and early 2025 that nonetheless left total abductions at 718 across the study period, with 34 persons killed. The article concluded that the persistent of kidnapping reflects not a failure of policy design, but a profound implementation gap rooted in five interrelated structural pathologies: fragmented inter-agency coordination, inadequate community intelligence integration, equipment and technological deficits, prosecutorial inefficacy, and the siloed intelligence culture among security institutions. The article recommended for prioritization of the Multi-Agency Anti-Kidnap Fusion Cell (MAAKFC), enforcement of cashless financial policies to disrupt ransom economies, and the formulation of a standalone national anti-kidnapping policy framework.

## Introduction

The Federal Capital Territory (FCT) of Nigeria occupies a unique and symbolically significant position in the country’s national security architecture. As the seat of federal governance, Abuja hosts key political institutions, diplomatic missions, and major economic activities, making its security central to the stability and image of the Nigerian state ( Federal Government of Nigeria, 2019). However, despite this strategic importance, the territory has increasingly experienced a rise in kidnapping incidents in recent years, raising serious concerns about the safety of residents and the effectiveness of security responses ( Nwosu et al., 2023). This trend persists even though national policy frameworks have identified kidnapping as a critical threat to national security ( ONSA, 2019).

Nigeria’s NSS, particularly the 2019 iteration and its 2023 update, articulates a whole-of-government approach to internal security, grounding anti-kidnapping efforts in intelligence-led operations, interagency coordination, and community partnership ( ONSA, 2019). Chapter 3, page 9 of the National Security Strategy (2019), titled “Armed Banditry, Kidnapping, Militancy and Separatist Agitations,” explicitly describes the criminal activities of armed bandits and kidnap gangs as the preeminent threats confronting us today, linking them to high economic pay-offs, the twin elements of fear and surprise, and the convergence of illegal networks. This rhetorical and formal recognition of the threat, however, has not translated into commensurate operational outcomes. Primary data collected by Beacon Security and Intelligence Limited on kidnapping incidents in the FCT between 2021 and 2025 documents 167 incidents resulting in 718 victims abducted and 34 killed, with yearly incident figures rising sharply from 2021 through 2023.

The persistence of this crisis invites a searching examination of the gap between policy formulation and operational effectiveness what scholars have characterized as a strategic-operational disconnect ( Bodunde & Balogun, 2018). This article addresses that gap by integrating three sources of evidence: an original incident-level dataset derived from open-source monitoring of FCT abduction events (2021–2025); primary data from interviews of security experts; and the rich theoretical and empirical frameworks developed in the security studies literature. The synthesis of these data sources yields an analysis that is simultaneously empirically grounded, theoretically coherent, and practically actionable.

## Literature review

### National security in contemporary context

National security has undergone significant conceptual evolution, reflecting profound shifts in the global security environment and changing state-society relations ( Baylis et al., 2017). Contemporary scholarship defines national security as the ability of a state to defend its sovereignty, territorial integrity, political institutions, and citizens from external threats and internal challenges through coordinated application of diplomatic, military, economic, and informational instruments of national power ( Hirsch Ballin et al., 2020). This multidimensional understanding emphasizes both internal and external security dimensions while recognizing the interrelationship between security, development, and governance.

The broadening of national security frameworks has incorporated human security dimensions, shifting focus from purely state-centric approaches to include protection of individuals and communities ( Hough et al., 2020). This paradigm emphasizes the preservation of basic human needs, social cohesion, and ecological integrity through governance structures that balance traditional security concerns with human security imperatives. The resilience-based approach to national security conceptualizes it as the capacity to endure, adjust to, and recover from complex disruptions while upholding fundamental values and maintaining essential functions ( Reznikova, 2023).

### Kidnapping as a security challenge

Kidnapping represents a complex criminal phenomenon with significant implications for national security and societal stability. Legally defined as the unlawful seizure, detention, or removal of an individual against their will through force, threat, or deception (Garner, 2019), kidnapping has evolved beyond a discrete criminal act to become a sophisticated security challenge with transnational dimensions. Contemporary conceptualizations emphasize kidnapping’s systemic roots, linking it to broader socio-economic disparities, political instability, and institutional weaknesses ( Okoli & Agada, 2014).

The multidimensional nature of kidnapping encompasses economic-motivated abductions focused on financial gain through ransom, political kidnapping aimed at advancing ideological goals, vendetta kidnapping stemming from personal conflicts, opportunistic kidnapping occurring alongside other criminal activities, and psychopathological kidnapping driven by psychological disorders ( Okoli & Agada, 2014). In the Nigerian context, kidnapping has emerged as a thriving criminal enterprise reflecting profound breakdown in societal order and deeper structural inequalities ( Enemuwe et al., 2026). The act serves not merely as a criminal event but as a manifestation of complex societal tensions, institutional failures, and economic marginalization ( Sheu & Ogunsola, 2025).

Theoretical frameworks for understanding kidnapping have evolved to incorporate routine activity theory, which posits that crime occurs when motivated offenders, suitable targets, and absence of capable guardians converge in time and space ( Cohen & Felson, 1979; Felson, 2008). Application of this framework to kidnapping in Nigeria reveals how socio-economic displacement creates motivated offenders, ostentatious wealth display identifies suitable targets, and weak law enforcement creates absence of capable guardians ( Akinyetun & Bakare, 2022).

### Security agency effectiveness and coordination

Effective security responses to complex threats like kidnapping require robust inter-agency coordination, intelligence integration, and community engagement ( Alemika, 2013). Intelligence-led policing frameworks position quality, timely, and actionable information as foundational to counter-kidnapping effectiveness ( Osolafia & Ugbedeojo, 2022). The establishment of specialized intelligence units, integration of technical and human intelligence sources, and development of analytical capacity represent critical institutional adaptations ( Fagbemi et al., 2024).

However, implementation challenges including resource constraints, institutional corruption, and community mistrust often undermine even well-designed strategic frameworks (Policy and Legal Advocacy Centre [PLAC], 2024). The literature emphasizes that fragmented security architectures with poor coordination mechanisms create operational gaps that criminal networks exploit ( Hameiri et al., 2018). The gap between policy formulation and operational reality represents a critical area requiring empirical investigation, particularly in contexts like Nigeria where multiple security agencies operate with overlapping mandates ( Alemika, 2013).

## Theoretical framework

### Routine activity theory

Routine Activity Theory (RAT), developed by ( Cohen & Felson, 1979), provides an analytically productive framework for understanding why kidnapping flourishes in particular temporal and spatial contexts. The theory argues that crime occurs when three conditions converge: the presence of a motivated offender, the availability of a suitable target, and the absence of capable guardianship. Applied to kidnapping in the FCT, the explanatory power of RAT is considerable.

Motivated offenders in the FCT context include criminal gangs primarily driven by financial gain, non-state armed groups (NSAGs) using abduction for operational funding and psychological coercion, and opportunistic actors emboldened by systemic impunity. The incident data analyzed in Figure 4 of this study confirms that criminals account for 107 of the 167 recorded incidents (64%), while NSAGs account for 40 (24%), and bandits for 11 (7%). This distribution underscores the predominantly economic character of kidnapping in the territory, consistent with the scholarly characterization of the Nigerian kidnapping economy as a self-sustaining criminal enterprise estimated to cost the country approximately $5 billion annually ( SBM Intelligence, 2023).

**Figure 1.**
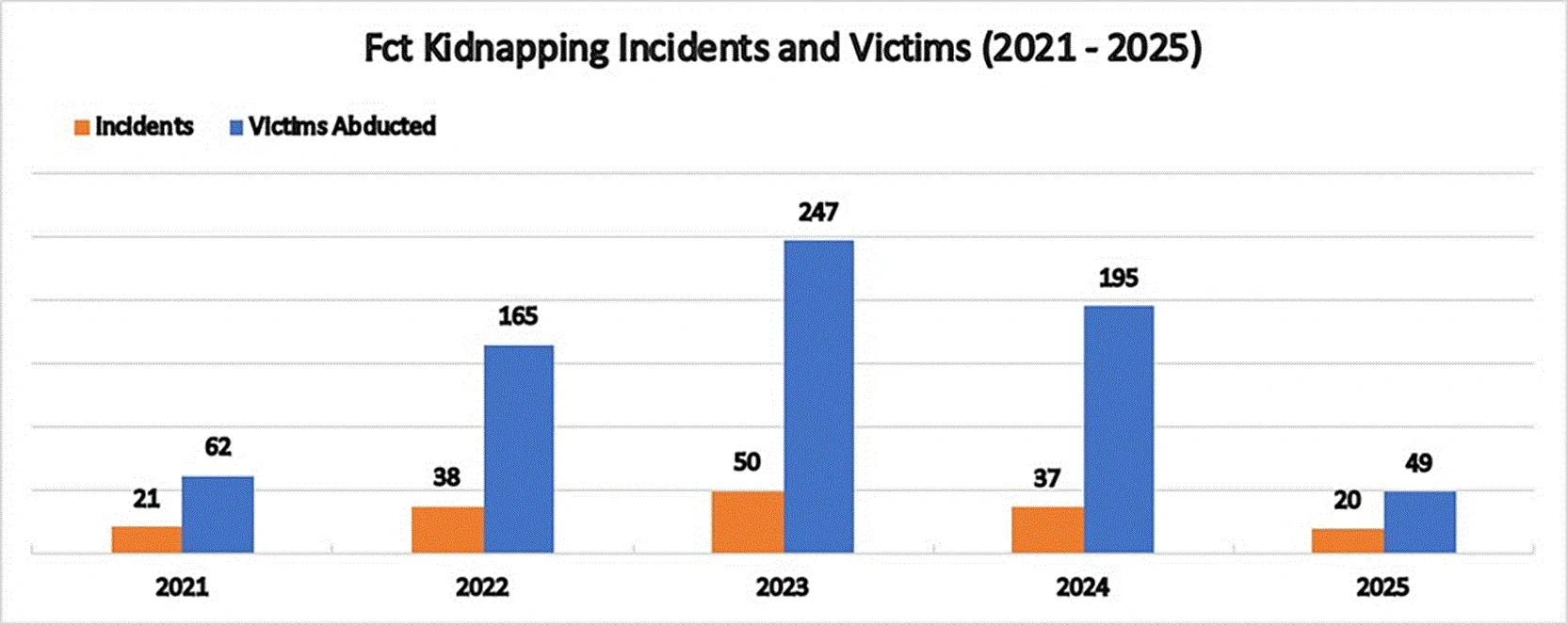
Annual Kidnapping Incidents and Victims by Year in the FCT (2021–2025). Figure 1 illustrates the escalating trajectory of FCT kidnapping incidents and victim counts from 2021 to early 2025. Incident frequency increased by 138% between 2021 and 2023 (from 21 to 50 per year), while total victims nearly quadrupled from 62 to 247. A partial numerical decline is observed in 2024, coinciding with the establishment of the MAAKFC in December 2024. Source: Beacon Security and Intelligence Limited Data, 2025.

**Figure 2.**
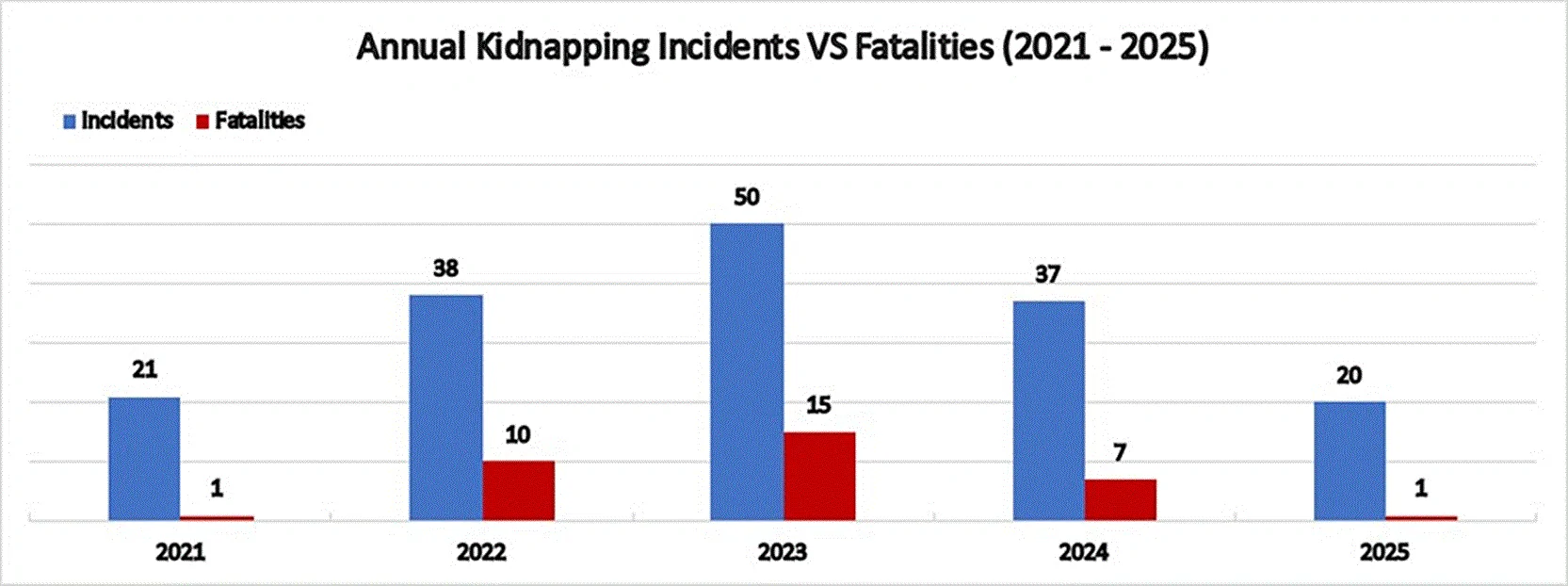
Annual Kidnapping Incidents versus Fatalities in the FCT (2021–2025). Figure 2 juxtaposes the annual volume of kidnapping incidents against recorded fatalities, illustrating the lethal dimension of the crisis alongside its frequency. The year 2023 recorded the highest number of both incidents (50) and fatalities (15), confirming it as the crisis apex of the study period. Source: Beacon Security and Intelligence Limited Data, 2025.

**Figure 3.**
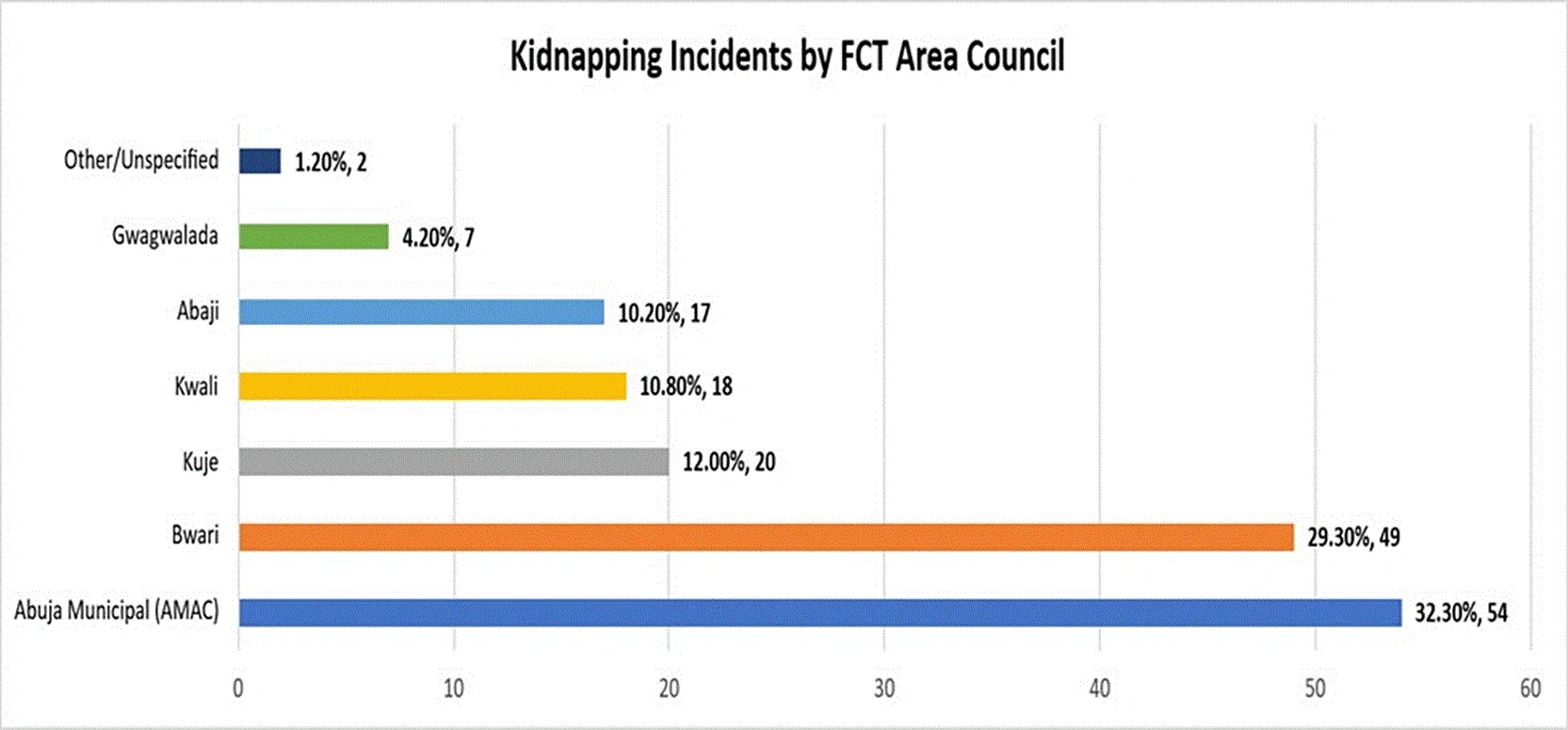
Kidnapping Incidents by FCT Area Council (2021–2025). Figure 3 ranks area councils by kidnapping incidence. Abuja Municipal Area Council (AMAC) and Bwari together account for 61.7% of all documented incidents. Bwari, Kuje, and Kwali are identified as the principal kidnapping hotspots, reflecting the spatial logic of criminal opportunity at peri-urban and rural fringe zones. Source: Beacon Security and Intelligence Limited Data, 2025.

**Figure 4.**
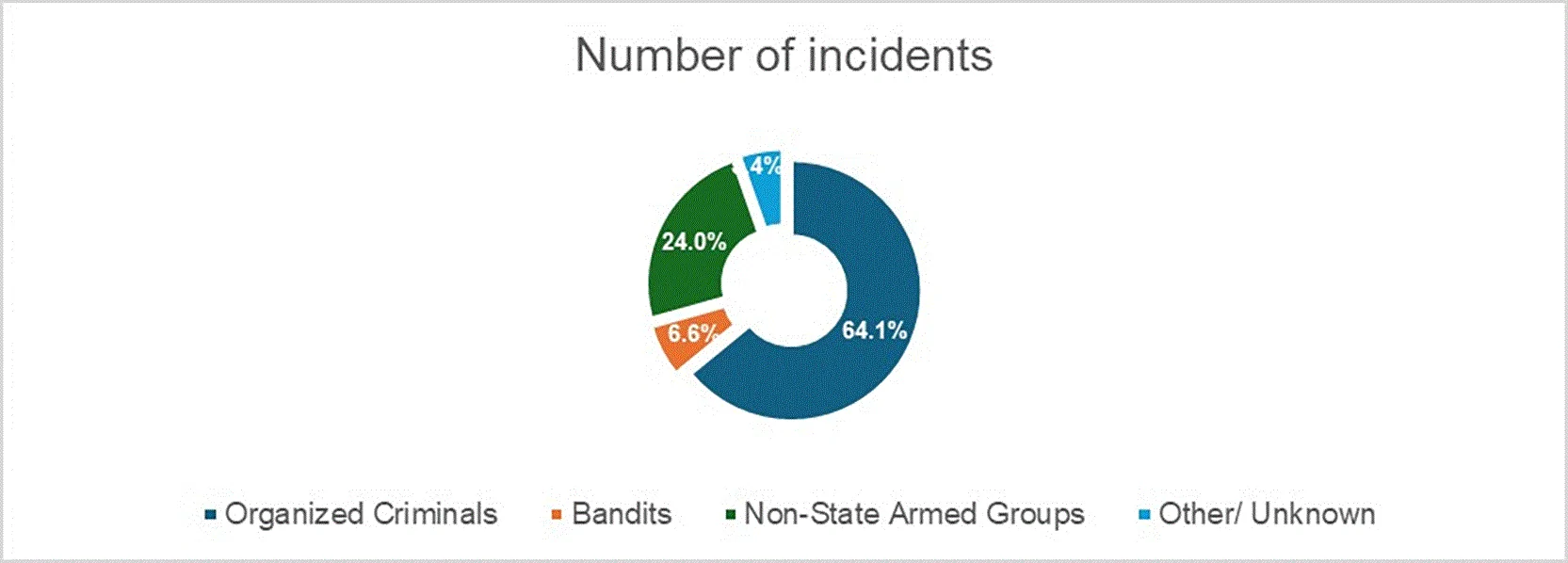
Distribution of Threat Actors in FCT Kidnapping Incidents (2021–2025). Figure 4 presents the distribution of threat actors responsible for kidnapping incidents in the FCT. Organized criminal groups account for 64.1% of all incidents, followed by Non-State Armed Groups (23.9%) and Bandits (6.6%). Source: Beacon Security and Intelligence Limited Data Analysis, 2025.

The absence of capable guardianship is the element most directly addressable through security policy. Here, RAT connects with institutional analysis: guardianship is absent not merely because security forces are physically unavailable, but because of inter-agency rivalries, equipment deficits, and the siloing of intelligence reduce the effective reach of security institutions. As the expert informant noted, “security agencies don’t harness community-based intelligence” and approach intelligence “in a siloed way”, creating structural voids that motivated offenders predictably exploit.

### Institutional capacity theory

Institutional Capacity Theory (ICT), drawing on foundational work by Grindle (1997), North (1990), and Andrews (2013), offers a complementary analytical lens. ICT identifies five dimensions of institutional capacity: human resources and leadership; organizational structures and processes; resource availability and management; legitimacy and community trust; and adaptive learning capacity. Applied to Nigeria’s security apparatus, ICT illuminates a pattern of compound institutional deficiency across all five dimensions, from insufficient specialized training and inter-agency coordination failures to equipment deficits and legitimacy erosion through complicity allegations.

Together, RAT and ICT provide powerful explanatory architecture. Where RAT identifies the situational conditions enabling kidnapping, ICT explains why the institutional responses designed to disrupt those conditions remain chronically inadequate. The nexus of these two frameworks, the convergence of motivated offenders with weak guardianship, itself a product of institutional capacity failures is the central analytical contribution of this study.

## Methodology

### Research design

This research utilized a concurrent mixed-methods design, integrating quantitative incidents level data with qualitative data derived from expert testimony by security stakeholders to explore the implementation gap between Nigeria’s National Security Strategy (2019) and its practice by Nigerian security agencies against the crime of kidnapping in the FCT. The quantitative aspect provided an empirically scale and scope for kidnapping crisis (2021-2025), while the qualitative aspect was carried out through interviews with security professionals which was used to explain the institutional and operational factors behind incidents patterns.

### Population and sampling

The quantitative sample consists of all logged kidnapping and abduction events that occurred within the FCT from Jan 2021 - Feb 2025 (N = 167). Stratified random sampling was conducted for the FCT area council, thereby organizing and comparing cases for AMAC, Bwari, Kuje, Kwali, Abaji, Gwagwalada with the comparative incidence between FCT’s urban, peri-urban, and rural fringe strata. The qualitative sample consists of five security practitioners recruited using a mixed purposive (initial referral) and snowball referral (subsequent contact of additional sources) methodology that drew from individuals in either the Nigerian Police Force (NPF), Nigerian Security and Civil Defence Corps (NSCDC) or as independent security consultants and practitioners with particular areas of expertise.

### Instruments of data collection

Quantitative data: Beacon Security & Intelligence Limited, a private security consulting firm collected the quantitative data (incident-level records). Records from Beacon Security & Intelligence Limited are collated using open source/media monitoring, ground investigations and validated incident reporting. The records are compiled according to date, geographic area of location (area council and site type), number of victims, number of fatalities and the categorisation of the threat-actor.

Qualitative data: Primary qualitative data was gathered via semi-structured interviews. The interviews were conducted in person or virtually, while using a guide which had been composed around the key themes of the study.

### Data analysis

Quantitative data were analyzed descriptively by Microsoft Excel using frequencies, percentages and year-on-year change, disaggregated over time by year, area council, threat type, incident location and month to discern temporal and spatial trends. In addition, qualitative data were analyzed through the application of thematic analysis. The practitioner evidence was coded using both the five dimensions of Institutional Capacity Theory and the three convergence conditions of Routine Activity Theory in order to explore, interpret and substantiate the quantitative results.

### FCT kidnapping trends (2021–2025)

**Figure 5.**
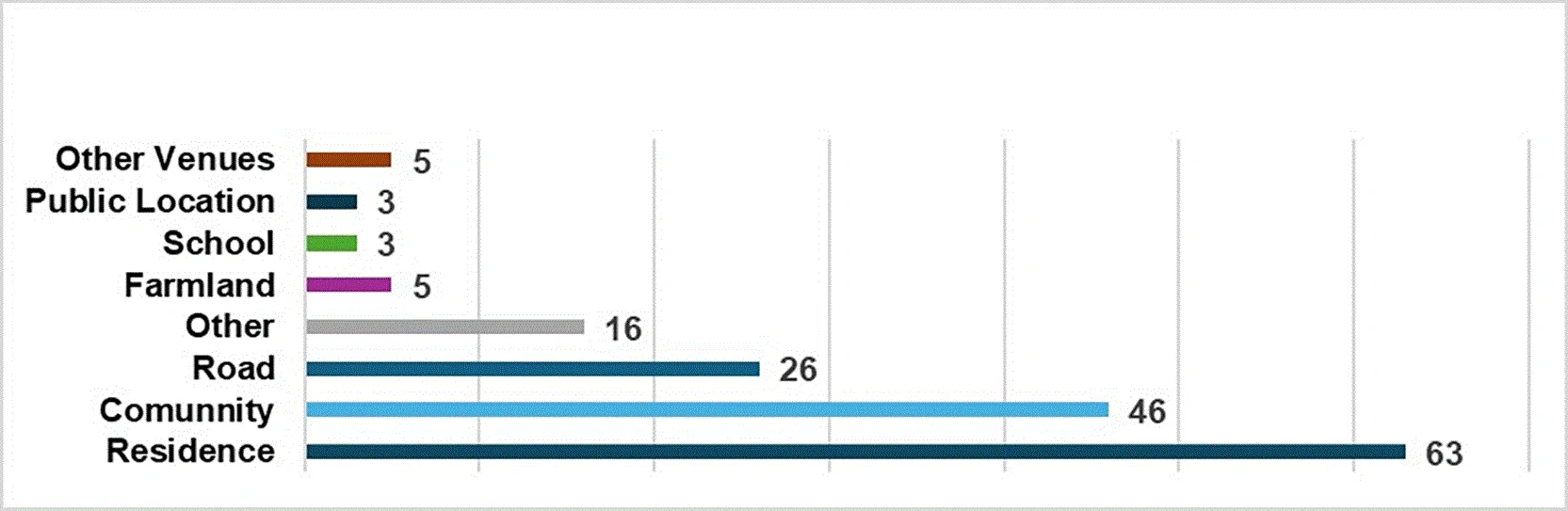
Kidnapping incidents by location type in the FCT (2021–2025). Figure 5 ranks kidnapping incidents by location type. Residences (63 incidents, 37.7%) and community spaces (46, 27.5%) together account for 65.3% of all incidents, underscoring the organized and intelligence-driven character of FCT abduction networks. Source: Beacon Security and Intelligence Limited Data, 2025.

**Figure 6.**
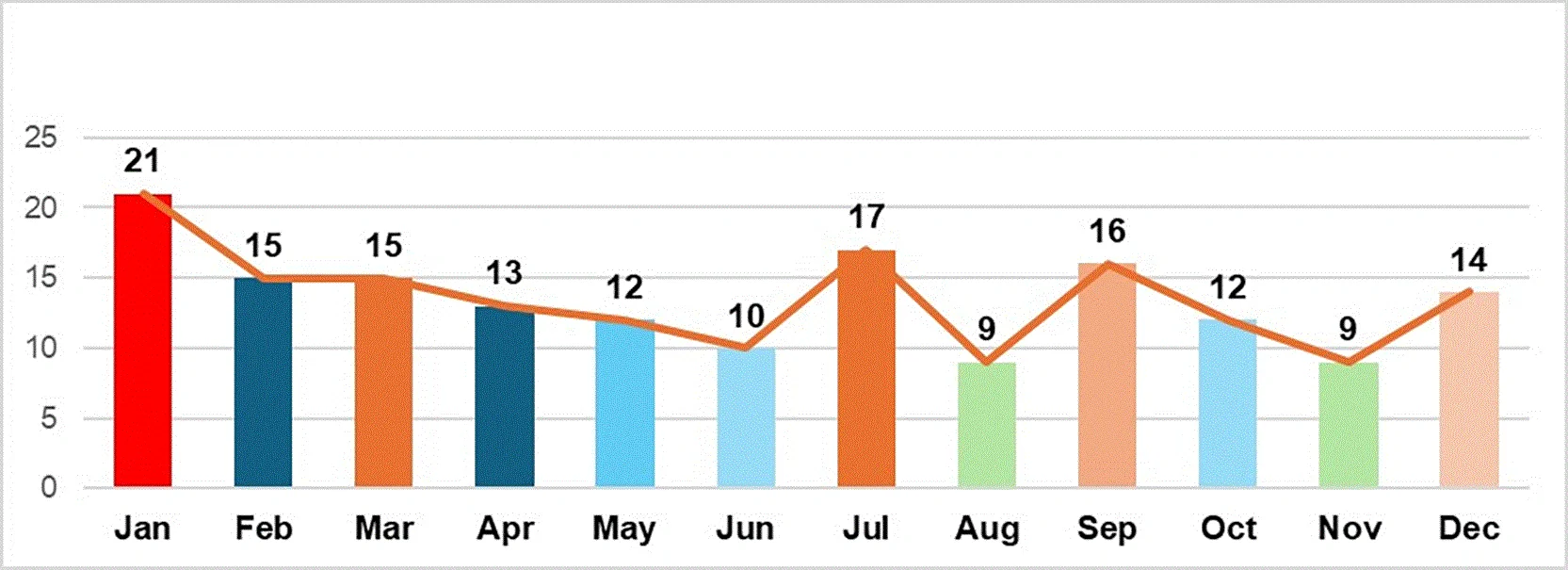
Monthly distribution of kidnapping incidents in the FCT (2021–2025). Figure 6 pools monthly incident counts across all years. January records the highest cumulative monthly total (21 incidents), consistent with post-holiday economic stress and reduced security alertness. The colour gradient (blue = lower frequency; red = higher frequency) highlights seasonal intensity patterns. August and November record the lowest monthly totals (9 each). Source: Beacon Security and Intelligence Limited Data, 2025.

Kidnapping in Nigeria’s Federal Capital Territory is not a story of isolated criminal incidents. It is a crisis that has metastasized steadily, more organized, and more lethal, penetrating a city that was designed to project national stability and sovereign authority. Between January 2021 and February 2025, 167 kidnapping and abduction events were recorded across the FCT, events that collectively claimed 718 victims and took 34 lives. Those numbers alone would be alarming. What makes them especially so is the trajectory they describe: from 21 incidents and 62 victims in 2021, the crisis escalated sharply to 50 incidents and 247 victims at its 2023 peak, an increase in frequency and a near-quadrupling of the human toll in just two years. Even the numerical decline observed in 2024 offers little reassurance; 37 incidents and 195 victims in a single year remain far beyond what any capital city should be expected to absorb. The data presented in Tables 1– 5 and Figures 1– 6 at the end of this article provide the full statistical picture; what follows is an analysis of what those numbers reveal about the character, geography, and drivers of the FCT kidnapping crisis.

**Table 1.** FCT Kidnapping incidents and victims by year (2021–2025).

Year	Incidents	Victims abducted	Fatalities	YoY change (%)
2021	21	62	1	-
2022	38	165	10	+81.0%
2023	50	247	15	+31.6%
2024	37	195	7	−26.0%
2025	20	49	1	N/A
*Total*	**166**	**718**	**34**	

Source: Beacon Security and Intelligence Limited Data, 2025.

**Table 2.** Kidnapping incidents by FCT area council (2021–2025).

Area council (LGA)	No. of incidents	% of total	Security profile
Abuja Municipal (AMAC)	54	32.3%	Urban Core
Bwari	49	29.3%	Peri-urban
Kuje	20	12.0	Peri-urban
Kwali	18	10.8%	Rural fringe
Abaji	17	10.2%	Rural fringe
Gwagwalada	7	4.2%	Satellite town
Other/Unspecified	2	1.2%	-

Source: Beacon Security and Intelligence Data, 2025.

**Table 3.** Threat actors.

Threat Actors	No. of incidents	Percentage
Organized criminals	107	64.1%
Bandits	11	6.6%
Non- state armed groups	40	24.0%
Other/Unknown	9	5.4%
	**167**	

Source: Beacon Security and Intelligence Limited Data, 2025.

**Table 4.** Kidnapping incidents by location type – FCT.

Resident	63
Community	46
Road	26
Other	16
Farmland	5
School	3
Public Location	3
Other Venues	5

Source: Beacon Security and Intelligence Limited Data, 2025.

**Table 5.** Monthly distribution of kidnapping incidents in the FCT.

January	21
February	15
March	15
April	13
May	12
June	10
July	17
August	9
September	16
October	12
November	9
December	14

Source: Beacon Security and Intelligence Limited Data, 2025.

### Temporal patterns and escalation

The incident dataset compiled from open-source security monitoring captures 167 documented kidnapping and abduction events in the FCT between January 2021 and early 2025. The data reveals a pronounced escalating trajectory across the study period, with both incident frequency and victim numbers rising sharply from 2021 through 2023 before a partial numerical moderation in 2024 and 2025 (see Table 1).

Figure 1 reveals an unmistakable escalation. Between 2021 and 2023, incident frequency increased by 138% (from 21 to 50 per year), while the total number of victims nearly quadrupled from 62 to 247. Fatalities rose in parallel, peaking at 15 in 2023. The partial decline in 2024 (37 incidents, 195 victims) coincides with the December 2024 establishment of the MAAKFC, though the absolute volume of abductions remains elevated.

Figure 2 emphasizes that the kidnapping crisis carries a deadly dimension often understated in incident counts alone. The year 2023 recorded not only the highest number of incidents (50) but also the most fatalities (15), confirming it as the unambiguous crisis apex of the study period. The 34 deaths across 167 incidents represent a case fatality rate of approximately 4.7% meaning that for every twenty incidents, at least one person was killed.

### Geographic distribution and hotspot analysis

Geographically, the data reveals a clear concentration of kidnapping activity in specific area councils (see Table 2 and Figure 3). Abuja Municipal Area Council (AMAC) records the highest absolute number of incidents (54), followed closely by Bwari (49), Kuje (20), Kwali (18), and Abaji (17). Gwagwalada accounts for 7 incidents. This distribution, however, must be read alongside the population and governance density of each area council: AMAC is the most urbanized and most heavily policed, which suggests either an incomplete picture driven by greater reporting rates in urban areas, or more significantly that kidnapping networks have penetrated the urban core of the FCT itself.

The identification of Bwari, Kuje, and Kwali as primary hotspots is consistent with expert assessment and existing quantitative data. Security experts have identified these three area councils as the major kidnapping hotspots within the FCT, attributing their vulnerability to a combination of peri-urban geography, porous interstate borders, and inadequate security infrastructure (Kabir, 2026). These zones sit at the intersection of urban and rural terrain precisely the spatial configuration that, under Routine Activity Theory, maximizes criminal opportunity by combining accessible targets with reduced guardianship. The revitalized G-7 joint task force comprising the FCT and its six neighboring states (Benue, Kaduna, Kogi, Nasarawa, Niger, and Plateau) was specifically designed to address the interstate border exploitation that characterizes kidnapping operations in this spatial zone.

The concentration in urban-fringe area councils Bwari (29.3%), Kuje (12.0%), Kwali (10.8%), and Abaji (10.2%) reflects the spatial logic of criminal opportunity theorized by Routine Activity Theory: these zones sit at the intersection of urban and rural terrain, combining accessible targets with reduced guardianship and exploitable interstate border corridors. The revitalized G-7 joint task force, comprising the FCT and six neighboring states (Benue, Kaduna, Kogi, Nasarawa, Niger, and Plateau), was specifically designed to address this border exploitation dynamic (Beacon Security, 2026).

### Location and threat actors

Analysis of incident location ( Figure 5) reveals that residences are the most common site of abduction (63 incidents, 37.7%), followed by communities (46, 27.5%) and road corridors (26, 15.6%). The preponderance of residential and community abductions is significant: it suggests that kidnapping networks are not merely opportunistic highway criminals, but organized actors with pre-operational intelligence about specific households and community gatherings. Threat actor data is presented in Table 3.

Threat actor analysis ( Figure 4) confirms that organized criminals account for the overwhelming majority of perpetrators (107 incidents, 64.1%), followed by non-state armed groups (40, 23.9%) and bandits (11, 6.6%). High-magnitude events including the abduction of 29 persons in Kwali (April 2023), 24 in Bwari (January 2024), and two separate mass abductions of 23 persons in Bwari within three months of each other (December 2023 and February 2024) point to highly organized criminal networks with sustained operational capacity. The two June 2022 incidents in AMAC involving alleged kidnappers in army and police uniforms respectively signal the threat of security force impersonation and potentially collusion, a dimension of institutional complicity documented consistently in the empirical literature ( Nwosu et al., 2023). Kidnapping incidents by location type are detailed in Table 4, while the monthly distribution of incidents is presented in Table 5.

Monthly distribution data ( Figure 6) reveals that January records the highest cumulative incident count across the study period (21), consistent with the post-holiday economic stress hypothesis and reduced security alertness at the start of the year. July also shows a notable peak (17 incidents), while August and November record the lowest totals (9 each). The three-month moving average confirms a relatively sustained level of threat throughout the year, with no pronounced “safe season,” indicating that kidnapping operations in the FCT are not seasonally determined but are a year-round structural phenomenon.

## Security agencies and the implementation of Nigeria’s national security strategy

### The normative framework: NSS mandates and agency responsibilities

Nigeria’s National Security Strategy establishes a normative framework within which security agencies are assigned complementary, if not always clearly delineated, anti-kidnapping responsibilities. The 2019 NSS, grounded in four core pillars security, prosperity, stability, and international cooperation, adopts a whole-of-government approach that formally recognizes the interrelationship between security, development, and governance. The primary agencies deployed against kidnapping in the FCT are the Nigerian Police Force (NPF), the Department of State Services (DSS), the Nigerian Security and Civil Defence Corps (NSCDC), the Nigerian Army through Operation Safe Haven, and various intelligence bodies including the Joint Intelligence Board (JIB) and the Intelligence Community Committee (ICC).

The expert assessment, however, offers a sobering corrective to this formal architecture. Asked to evaluate the operational effectiveness of security agencies in terms of response time, rescue operations, and disruption of kidnapping networks, security experts are of the opinion that the security agencies have been largely ineffective majorly because of the absence of a coordinated and centralized command structure. This encapsulates the central empirical finding of this article: that formal mandates, legal frameworks, and coordination mechanisms prescribed by the NSS are not translating into operational effectiveness on the ground.

### The multi agency anti-kidnap fusion cell (MAAKFC)

A notable development in the federal government’s evolving response to kidnapping was the establishment of the Multi-Agency Anti-Kidnap Fusion Cell (MAAKFC) on December 19, 2024, with the support of the United Kingdom’s National Crime Agency (NCA). The MAAKFC is a specialized unit domiciled in the Office of the National Security Adviser (ONSA), specifically within the National Centre for Counter Terrorism. Its mandate is to coordinate national security agencies in combating kidnapping across Nigeria through enhanced intelligence sharing, operational coordination, and joint rapid responses a formal institutional shift from reactive to proactive, intelligence-led operations.

This development is analytically significant on two counts. First, it represents an implicit acknowledgement by the federal government that the pre-existing multi-agency architecture was inadequate that the jurisdictional fragmentation and intelligence siloing identified in this, and other studies required institutional remedy at the highest level of the security establishment. Second, the placement of MAAKFC within the ONSA reflects a centralization logic consistent with the NSS’s emphasis on the National Security Adviser as the apex coordinator of anti-kidnapping efforts. The UK-NCA technical partnership further signals Nigeria’s recognition that domestic institutional capacity requires international augmentation.

However, the expert assessment cautions against premature optimism. The MAAKFC’s effectiveness remains challenged, with evidence that kidnapping for ransom is ongoing (The data partially supports this: while 2024 shows a reduction in incidents compared to 2023’s peak, the absolute volume of abductions remains high (37 incidents, 195 victims), and MAAKFC was only operational for the final weeks of 2024. Its institutional effectiveness over a sustained period remains to be demonstrated.

## Structural and institutional challenges undermining anti-kidnapping operations

### Intelligence siloing and inter-agency rivalry

The most persistently documented structural challenge to security effectiveness in the FCT is the fragmentation of intelligence across agencies with competing institutional cultures and mandates. Security experts confirm directly that coordination gaps and institutional rivalries exist, characterizing security organizations as seeing intelligence in a siloed way. The silo dynamic is particularly damaging in the kidnapping context because anti-kidnapping operations are inherently cross-jurisdictional: abductors typically strike in one area council, hold victims in another, and demand ransoms through yet another node of criminal network activity. Effective response requires near-real-time intelligence exchange between the NPF, DSS, NSCDC, military intelligence, and state-level agencies. When each institution guards its intelligence as a proprietary asset, the seams between institutional mandates become the operational corridors through which criminals move freely.

### Community intelligence

Community-based intelligence is widely recognized as one of the most cost-effective and contextually sensitive inputs into counter-kidnapping operations. Yet expert assessment is unambiguous: “Security agencies don’t harness community-based intelligence”. This failure has a dual consequence. First, it denies security agencies the actionable, granular intelligence that could enable preemptive disruption of kidnapping networks consistent with the RAT prediction that capable guardianship disrupts the convergence of offenders and targets. Second, it erodes the legitimacy of security agencies in the eyes of communities, who perceive institutional indifference as incompetence or collusion. The resulting legitimacy deficit creates a vicious cycle in which communities become less willing to share information, further degrading the intelligence base available to security agencies ( Rothstein, 2011; Agbiboa, 2015).

### Equipment deficits and technological gaps

Equipment deficits is one of the two primary structural constraints on security agency effectiveness in combating kidnapping. The NSS calls for technology adoption, yet this aspiration collides with the realities of chronic underfunding, procurement failures, and the rapid technological sophistication of kidnapping networks. Modern kidnapping operations increasingly employ digital tracking systems, encrypted communications platforms, and cybercrime methods for laundering ransom payments to evade detection ( Enemuwe et al., 2026). The asymmetry between the technological capabilities of criminal networks and those of state security institutions is one of the most consequential manifestations of institutional capacity failure in the FCT.

### Prosecutorial failure and the impunity economy

Research consistently identifies prosecution as a potentially effective deterrent against kidnapping. Yet, research observes that, prosecution is rare in the FCT, making it difficult to demonstrate deterrent effect in the local context. The resulting impunity economy in which kidnapping generates large financial rewards at low legal risk creates the precise conditions under which RAT predicts criminal activity to flourish. The weakness of prosecution reflects upstream failures of investigation and evidence collection, which are themselves products of intelligence deficits and coordination failures.

### Policy implications and the case for a standalone anti-kidnapping framework

The cumulative weight of the evidence reviewed in this article supports a clear policy conclusion: Nigeria requires a standalone national anti-kidnapping policy framework, separate from the broader NSS. Given how pervasive the threat of kidnapping for ransom is. Also, the current approaches are fragmented. The existing policy architecture which treats kidnapping as one element within a broader security strategy does not provide the dedicated institutional focus, resource allocation specificity, or performance accountability mechanisms that the scale of the kidnapping crisis demands.

A standalone framework would need to: codify the mandate and resourcing of the MAAKFC with statutory authority; establish minimum standards for inter-agency intelligence sharing with defined legal consequences for non-compliance; create community intelligence integration protocols; and establish prosecutorial performance metrics that create institutional accountability for criminal justice outcomes. On the financial disruption dimension, ensuring the effectiveness of the cashless policy and monitoring cash movements in the shadow economy as a primary strategic intervention recognizing that kidnapping is ultimately a financial enterprise whose economic viability can be disrupted through targeted financial intelligence operations.

## Conclusion

This article has demonstrated, through the convergence of incident-level data, expert testimony, and theoretical analysis, that the kidnapping crisis in Nigeria’s Federal Capital Territory is not a consequence of policy ignorance, but of policy implementation failure at multiple institutional levels. Between 2021 and 2025, 167 documented incidents resulted in 718 persons abducted and 34 killed a trajectory of escalation that exposes the gap between the aspirations of the National Security Strategy and the operational realities of the security agencies mandated to implement it.

The analytical framework developed in this article integrating Routine Activity Theory’s focus on situational conditions enabling crime with Institutional Capacity Theory’s focus on organizational determinants of security effectiveness yields a coherent explanation of this failure. Motivated offenders have exploited the absence of capable guardianship created by fragmented inter-agency coordination, community intelligence neglect, technological deficits, and prosecutorial weakness. The geographic concentration of abductions in Bwari, Kuje, and Kwali reflects the spatial logic of criminal opportunity: these peri-urban and rural fringe areas combine accessible targets with reduced guardianship and exploitable interstate border corridors.

The establishment of the MAAKFC in December 2024 represents a genuine, if belated, institutional response to these structural failures. However, the evidence reviewed here suggests that its effectiveness will depend on sustained resourcing, statutory authority, and a genuine cultural change within security institutions towards collaborative, intelligence-sharing operational practice. Without these conditions, MAAKFC risks becoming another formal mechanism that, like its predecessors, fails to bridge the strategic-operational gap that has defined Nigeria’s anti-kidnapping efforts since 2019. The deeper implication of this study is that kidnapping in the FCT demands a dedicated policy architecture a standalone national anti-kidnapping framework with the institutional coherence, resource specificity, and accountability mechanisms proportionate to a threat estimated to cost Nigeria $5 billion annually ( SBM Intelligence, 2023). The current approach by security agencies is disjointed and the data confirms this assessment with unambiguous clarity.

### Recommendations

Drawing from empirical analysis, expert testimony, and theoretical frameworks, this article advances six interconnected recommendations:

(i) Institutionalize and Fully Resource the MAAKFC: The Multi-Agency Anti-Kidnap Fusion Cell must be given statutory authority, dedicated budget lines, and clear performance accountability mechanisms. Equipping the MAAKFC with advanced intelligence analysis tools, real-time data sharing platforms, and joint operational capacity is the most immediate structural intervention available within the existing institutional architecture.
Implementing Agency: Office of the National Security Adviser (ONSA), in conjunction with the National Assembly (for statutory authority) and the Federal Ministry of Finance (for dedicated budget allocation).
(ii) Establish a Standalone National Anti-Kidnapping Policy Framework: A dedicated policy instrument separate from the NSS is needed to consolidate mandates, close coordination gaps, and create a unified accountability structure for anti-kidnapping operations across all relevant agencies and tiers of government.
Implementing Agency: ONSA, in collaboration with the Federal Ministry of Justice and the National Assembly.
(iii) Deploy Cashless Policy Enforcement and Shadow Economy Monitoring: Financial intelligence agencies must work in concert with the MAAKFC to monitor high-volume cash transactions, target the hawala and informal value transfer networks through which ransoms are paid, and disrupt the financial infrastructure of kidnapping networks.
Implementing Agency: Central Bank of Nigeria (CBN) and the Nigerian Financial Intelligence Unit (NFIU), working in concert with the MAAKFC.
(iv) Formalise Community Intelligence Integration: Security agencies must develop structured, resourced, and legally protected community intelligence collection systems. This requires not only protocol development but also a fundamental cultural shift within security institutions away from intelligence siloing and towards trust-based community engagement.
Implementing Agency: Nigerian Police Force (NPF) and NSCDC at the FCT Command level, with FCT Administration support for community liaison structures.
(v) Prioritise Prosecution: Anti-kidnapping units must be matched by dedicated prosecutorial capacity. This includes trained prosecutors with security clearances, specialised courts with accelerated procedures for kidnapping cases, and robust witness protection programmes that enable victims and witnesses to cooperate with the criminal justice system without fear of reprisal.
Implementing Agency: Federal Ministry of Justice and the National Judicial Council (for specialized courts), with the FCT Attorney-General’s office as primary implementer.
(vi) Accelerate Technology Modernisation: Security agencies must be equipped with geospatial crime mapping tools, AI-assisted surveillance systems, encrypted inter-agency communication platforms, and digital forensics capabilities sufficient to counter the technological advantages currently enjoyed by sophisticated kidnapping networks.
Implementing Agency: NPF, DSS, and NSCDC procurement/ICT departments, with technical partnership support from ONSA’s MAAKFC and international partners such as the UK’s National Crime Agency.

## Data Availability

The aggregated dataset underlying this study is openly available in figshare at https://doi.org/10.6084/m9.figshare.32666499 ( Yahaya & Garba, 2026). It contains the yearly totals, area-council distributions, threat-actor breakdowns, location-type frequencies, and monthly distributions for the 167 documented kidnapping and abduction events recorded in Nigeria’s Federal Capital Territory between January 2021 and February 2025, as compiled by Beacon Security and Intelligence Limited. These aggregated values underpin all means, percentages, tables ( Tables 1– 5), and figures ( Figures 1– 6) reported in this article and are released under a CC BY 4.0 licence. The complete raw, incident-level records cannot be made publicly available because they contain sensitive security information, victim identities, and operational intelligence whose disclosure would breach privacy and security obligations. Access to the raw dataset may be requested from Beacon Security and Intelligence Limited, subject to their confidentiality and data-sharing conditions.
