# Supplementary material for: NIGERIA'S NATIONAL SECURITY STRATEGY AND THE ROLE OF SECURITY AGENCIES IN COMBATTING KIDNAPPING IN THE FEDERAL CAPITAL TERRITIORY ABUJA FROM 2021-2025
Source: Open Res Afr. 2026 Sep 3;9:40. Originally published 2026 Jul 6. [Version 2] doi: 10.12688/openresafrica.16586.2 (PMC13562926; doi:10.12688/openresafrica.16586.2)
Supplement: Peer review history

# Reviewer report: Reviewer response for version 2

**DOI:** 10.21956/openresafrica.18105.r36088

**Published:** 2026-09-12

**Authors:** Ashibi Jacob

**Affiliations:** 1 Kampala International University, Kampala, Uganda

The research article has now been significantly improved and passed my initial assessment.

Is the work clearly and accurately presented and does it cite the current literature?

Yes

If applicable, is the statistical analysis and its interpretation appropriate?

Yes

Are all the source data underlying the results available to ensure full reproducibility?

Yes

Is the study design appropriate and is the work technically sound?

Partly

Are the conclusions drawn adequately supported by the results?

Yes

Are sufficient details of methods and analysis provided to allow replication by others?

Yes

Reviewer Expertise:

NA

I confirm that I have read this submission and believe that I have an appropriate level of expertise to confirm that it is of an acceptable scientific standard.

# Reviewer report: Reviewer response for version 2

**DOI:** 10.21956/openresafrica.18105.r36106

**Published:** 2026-09-10

**Authors:** Oluwatoyin Olatundun ILESANMI

**Affiliations:** 1 Redeemer's University, Ede, Nigeria

**SUMMARY OF THE ARTICLE - NIGERIA'S NATIONAL SECURITY STRATEGY AND THE ROLE OF SECURITY AGENCIES IN COMBATTING KIDNAPPING IN THE FEDERAL CAPITAL TERRITORY ABUJA FROM 2021-2025**

 This article investigates the escalating crisis of kidnapping in Nigeria’s Federal Capital Territory (FCT) between 2021 and 2025. It utilizes a concurrent mixed-methods design, combining an original dataset of 167 kidnapping incidents (sourced from Beacon Security & Intelligence Limited) with qualitative interviews from security experts. Guided by Routine Activity Theory (RAT) and Institutional Capacity Theory (ICT), the study evaluates the effectiveness of Nigeria’s 2019 National Security Strategy (NSS). The authors find that despite the NSS framework, kidnapping has increased due to a profound implementation gap characterized by intelligence siloing, fragmented inter-agency coordination, technological deficits, and prosecutorial failures. The article concludes by recommending the institutionalization of the Multi-Agency Anti-Kidnap Fusion Cell (MAAKFC) and the creation of a standalone national anti-kidnapping policy

**DETAILED REVIEW AND MAJOR COMMENTS**

1. **Methodological Accuracy (Quantitative Sampling):** The methodology section describes the quantitative data as follows: "Stratified random sampling was conducted for the FCT area council... organizing and comparing cases for AMAC, Bwari, etc."
  - **Critique**: Since the dataset comprises all logged kidnapping events in the FCT from 2021–2025 (N=167), this is a census, not a sample. There is no "sampling" occurring here. The authors are simply organizing (stratifying) the population of recorded incidents. Using the term "random sampling" is misleading. I recommend revising the language to state that the study utilized a census of recorded incidents organized by area council stratification.
2. **Causality vs. Correlation (MAAKFC): **The text states: "The partial decline in 2024... coincides with the December 2024 establishment of the MAAKFC."
  - **Critique:** This phrasing risks implying a causal relationship between the establishment of the MAAKFC (Dec 2024) and the decline in incidents throughout 2024. Attributing the general downward trend observed throughout 2024 to the Multi-Agency Anti-Kidnap Fusion Cell (MAAKFC) is temporally incongruous, given that the agency was not operational until the final month of the year. The decline in 2024 likely began before the MAAKFC was operational. The authors should temper this claim to avoid a post hoc fallacy. The 2024 data likely reflect pre-MAAKFC trends or other factors.
3. **Data Source Limitations:** Reliance on a single private security firm (Beacon Security & Intelligence Limited) as the sole source for the entire quantitative dataset (N=167) introduces a potential risk of selection bias or incompleteness compared to official state records (which may be underreported). 
  - **Critique**: While official data is often unreliable, the manuscript would benefit from a more explicit discussion in the limitations section regarding why this private source was chosen over others and the potential limitations of relying on a proprietary dataset.
4. **Qualitative Rigour: **The qualitative sample consists of only five (5) individuals.
  - **Critique: **While expert interviews can be small, N=5 is a very limited base for generalizing about the entire institutional failure of the Nigerian security apparatus. The authors should provide more detail on the "thematic analysis" process. For instance, was the analysis inductive or deductive? Was software used? How was inter-coder reliability ensured? Expanding on the analysis section would strengthen the trustworthiness of the qualitative findings.
5. **Minor Comment**
  - ** Typographical/Grammatical Errors**: Page 4: "The persistent of kidnapping..." should be "The persistence of kidnapping..."
  - **Theoretical Application: **The application of Routine Activity Theory is strong. However, the application of Institutional Capacity Theory could be deepened. The five dimensions are listed, but the analysis could explicitly map each of the five "structural pathologies" identified (e.g., fragmentation, equipment deficits) directly to the specific dimensions of ICT (e.g., resource availability, organizational structures) to tighten the theoretical argument.

**Conclusion**

 This is a timely and relevant study that addresses a critical national security issue. The integration of quantitative trends with expert qualitative insight offers valuable contributions to the literature. The recommendations are practical and policy oriented. The scientific rigor of the manuscript would be significantly improved by correcting the sampling terminology, qualifying causal claims related to the MAAKFC, and providing greater detail on the qualitative analysis. I recommend indexing after these revisions are made.

 The authors need to correct the comments raised and also clean up the references. For instance, page 12: References list contains a future date citation (Enemuwe et al., 2026). While the article period focus range 2021-2025. The Authors need to ensure all citations are accurate and verified.

Is the work clearly and accurately presented and does it cite the current literature?

Yes

If applicable, is the statistical analysis and its interpretation appropriate?

Yes

Are all the source data underlying the results available to ensure full reproducibility?

Yes

Is the study design appropriate and is the work technically sound?

Yes

Are the conclusions drawn adequately supported by the results?

Yes

Are sufficient details of methods and analysis provided to allow replication by others?

Partly

Reviewer Expertise:

Clinical & Environmental Psychology, Mental Health, Gender, Social Policy, Humanitarianism and Development

I confirm that I have read this submission and believe that I have an appropriate level of expertise to confirm that it is of an acceptable scientific standard, however I have significant reservations, as outlined above.

# Reviewer report: Reviewer response for version 1

**DOI:** 10.21956/openresafrica.17778.r35224

**Published:** 2026-08-06

**Authors:** Ashibi Jacob

**Affiliations:** 1 Kampala International University, Kampala, Uganda

The manuscript addresses a highly relevant and timely issue, examining the challenge of insecurity within the Federal Capital Territory (FCT) and its broader implications for Nigeria. The research problem is clearly articulated, providing a strong foundation for the study.

 The literature review is comprehensive, current, and relevant to the subject matter. Furthermore, the theoretical framework is appropriate, with the selected theories effectively underpinning the study and providing a sound basis for the analysis.

 However, the methodological section requires significant improvement. The research design is insufficiently described, while the discussion of data sources and methods of data collection is skeletal and lacks the level of detail expected in an academic study. Greater methodological clarity is necessary to enhance the transparency and replicability of the research.

 In addition, the study relies excessively on a single data source, namely Beacon Security and Intelligence Limited. This overreliance weakens the robustness and credibility of the findings and increases the risk of drawing premature conclusions and making broad generalisations. To strengthen the empirical foundation of the study, the researchers should incorporate data from multiple authoritative sources, such as the Nigeria Police Force, the National Bureau of Statistics (NBS), the National Crime and Safety Survey, and the Federal Capital Territory Administration (FCTA). Triangulating data from these sources would improve the validity and reliability of the analysis.

 The presentation of the data also requires improvement. Specifically, the source of the data presented in each table should be clearly identified and appropriately referenced to enhance transparency and academic rigour.

 Finally, although the recommendations are relevant and potentially impactful, they lack specificity regarding implementation. The authors should clearly identify the institutions or stakeholders responsible for implementing each recommendation. This would enhance the practical relevance and policy value of the study.

Is the work clearly and accurately presented and does it cite the current literature?

Yes

If applicable, is the statistical analysis and its interpretation appropriate?

Yes

Are all the source data underlying the results available to ensure full reproducibility?

Yes

Is the study design appropriate and is the work technically sound?

Partly

Are the conclusions drawn adequately supported by the results?

Yes

Are sufficient details of methods and analysis provided to allow replication by others?

Yes

Reviewer Expertise:

Criminology and Security Studies

I confirm that I have read this submission and believe that I have an appropriate level of expertise to confirm that it is of an acceptable scientific standard, however I have significant reservations, as outlined above.

# Response

**Published:** 2026-08-25

**Authors:** Aisha Annie Yahaya

**Affiliations:** Political Science and International Relations, Nile University of Nigeria, Abuja, Federal Capital Territory, Nigeria

We thank the reviewer for the constructive feedback and the "approved with reservations" assessment. We have expanded the Methodology section to detail the research design, population, sampling technique, and data collection instruments for both the quantitative incident dataset and the qualitative interviews. We have acknowledged the single-source data limitation directly and clarified how triangulation across the five interview respondents, drawn from three distinct institutional vantage points, supports the dataset's credibility. Source attributions have been added to Tables 1–5, and each of the six policy recommendations now specifies the implementing agency responsible.

# Reviewer report: Reviewer response for version 1

**DOI:** 10.21956/openresafrica.17778.r35225

**Published:** 2026-08-06

**Authors:** Vivian Aziengbe

**Affiliations:** 1 Nasarawa State University, Keffi, Nasarawa, Nigeria

1. Yes, the work is closely and accurately presented as it covers 2021-2025, makes it valuable to studies on security in Nigeria. The literatures cited are current and needs to be critically analyzed rather than just making reference to their contributions.
2.  Yes, the study design is appropriate because it is mixed method. However, the work needs to be technically sound by providing more details on the data gathering methods such as sampling techniques and interview schedules. The work should show how reliability and validity measures were carried out.
3. No. The research methods and analysis are not sufficiently executed. The work should extensively define the research design methods, population of the study and sampling techniques with the use of data analysis software.
4. Yes, the statistical analysis and interpretation is excellent. The combination of statistics, figures, theory and other evidence gave the work a robust delivery.
5. Yes, all sources are captured in the reference. However, the references need to be proofread to ensure consistency with the adopted style.
6. Yes, the conclusions are adequately supported.

Is the work clearly and accurately presented and does it cite the current literature?

Yes

If applicable, is the statistical analysis and its interpretation appropriate?

Yes

Are all the source data underlying the results available to ensure full reproducibility?

Yes

Is the study design appropriate and is the work technically sound?

Yes

Are the conclusions drawn adequately supported by the results?

Yes

Are sufficient details of methods and analysis provided to allow replication by others?

No

Reviewer Expertise:

Communication and Media Studies

I confirm that I have read this submission and believe that I have an appropriate level of expertise to confirm that it is of an acceptable scientific standard.

# Response

**Published:** 2026-08-25

**Authors:** Aisha Annie Yahaya

**Affiliations:** Political Science and International Relations, Nile University of Nigeria, Abuja, Federal Capital Territory, Nigeria

We thank the reviewer for the positive assessment and helpful suggestions. We have strengthened the Methodology section with full detail on sampling technique, the interview schedule, and the reliability and validity measures applied to both the quantitative and qualitative data, addressing the replicability concern by specifying the research design, population, and analysis software used. Finally, we have proofread the reference list to ensure full consistency with the adopted citation style.
